# Molecular Mechanisms and Applications of N-Acyl Homoserine Lactone-Mediated Quorum Sensing in Bacteria

**DOI:** 10.3390/molecules27217584

**Published:** 2022-11-04

**Authors:** Lokender Kumar, Sanjay Kumar Singh Patel, Kusum Kharga, Rajnish Kumar, Pradeep Kumar, Jessica Pandohee, Sourabh Kulshresha, Kusum Harjai, Sanjay Chhibber

**Affiliations:** 1 School of Biotechnology, Faculty of Applied Sciences and Biotechnology, Shoolini University, Solan 173229, India; 2 Department of Chemical Engineering, Konkuk University, Seoul 05029, Korea; 3 Department of Pharmaceutical Engineering and Technology, Indian Institute of Technology, Banaras Hindu University, Varanasi 221005, India; 4 Telethon Kids Institute, Nedlands, WA 6009, Australia; 5 Department of Microbiology, Panjab University, Sector 14, Chandigarh 160014, India

**Keywords:** acyl-homoserine lactone, bacteria adaptation, biofouling, biomolecules, bioremediation, biosensor, cancer, human health, plant disease, quorum sensing

## Abstract

Microbial biodiversity includes biotic and abiotic components that support all life forms by adapting to environmental conditions. Climate change, pollution, human activity, and natural calamities affect microbial biodiversity. Microbes have diverse growth conditions, physiology, and metabolism. Bacteria use signaling systems such as quorum sensing (QS) to regulate cellular interactions via small chemical signaling molecules which also help with adaptation under undesirable survival conditions. *Proteobacteria* use acyl-homoserine lactone (AHL) molecules as autoinducers to sense population density and modulate gene expression. The LuxI-type enzymes synthesize AHL molecules, while the LuxR-type proteins (AHL transcriptional regulators) bind to AHLs to regulate QS-dependent gene expression. Diverse AHLs have been identified, and the diversity extends to AHL synthases and AHL receptors. This review comprehensively explains the molecular diversity of AHL signaling components of *Pseudomonas aeruginosa*, *Chromobacterium violaceum*, *Agrobacterium tumefaciens*, and *Escherichia coli*. The regulatory mechanism of AHL signaling is also highlighted in this review, which adds to the current understanding of AHL signaling in Gram-negative bacteria. We summarize molecular diversity among well-studied QS systems and recent advances in the role of QS proteins in bacterial cellular signaling pathways. This review describes AHL-dependent QS details in bacteria that can be employed to understand their features, improve environmental adaptation, and develop broad biomolecule-based biotechnological applications.

## 1. Introduction

Quorum sensing (QS) is a biochemical communication system in bacteria that directs social interactions based on population density [1,2]. Bacteria produce extracellular signals early in their adaptation and growth phase; as the cell density increases, the signals accumulate in the environment [3]. Once a critical cell density threshold is reached, these signals interact with their cognate receptors and coordinate the expression of associated genes [4]. In Gram-positive and Gram-negative bacteria, several QS systems have been identified [5,6], including acyl-homoserine lactone (AHL)-based and peptide-based QS systems. In addition, many proteobacteria use the AHL-dependent QS system to regulate population-dependent behaviors, including plasmid conjugation, pigment production, virulence factor expression, and biofilm formation [7]. In the late 1960s and early 1970s, AHL-QS was first discovered in a marine bacterium called *Vibrio fisheri* [8]. As part of its symbiotic relationship with the Hawaiian bobtail squid, *Euprymna scolopes, V. fisheri* regulates its bioluminescence in a population density-dependent manner. In the *V. fisheri* QS system, autoinducers (AIs) and two essential proteins (LuxR and LuxI) regulate bioluminescence signal production. The LuxI enzyme produces the QS signal molecule (the N-3-oxo-hexanoyl-homoserine lactone), and LuxR is a transcription regulator that binds to the AHL signal and regulates bioluminescence via the luxCDABEG operon [9]. Following the discovery of the AHL-QS system, researchers discovered that AHL-QS is prevalent in a wide range of *Proteobacteria* and influences vital characteristics such as motility [10], pigment production [11], and biofilm formation [12]. Our molecular understanding of the QS system has been useful in studying plant–microbe, animal–microbe, and human–microbe interactions.

The AHL molecule comprises a homoserine lactone ring and an acyl chain ranging from C4 to C18 in length [13]. Depending upon the organisms, the AHL molecules may have 3-hydroxy, 3-oxo, methyl, or varying unsaturation in some cases [14]. The first essential part of AHL signaling is the LuxI-type AHL synthases, which are involved in producing AHL molecules. Once the AHL molecules are generated, they can be transported passively and actively in and out of the cells [15]. The second component of AHL signaling is the LuxR-type AHL receptor proteins, which bind to the AHL signal molecule and govern the QS-dependent activation, repression, and depression of target genes [16,17]. The LuxR-type DNA-binding transcription regulators govern QS-dependent gene expression. Several reviews have summarized the molecular mechanism and phenotype regulation of AHL-QS systems. Still, there are few detailed structural and functional investigations of AHL-QS-associated proteins at the molecular level. Churchill et al. (2011) reviewed AHL signaling in exceptional detail and evaluated the molecular basis of AHL signaling in bacteria [18]. However, recent advancements in AHL-QS and the availability of new crystal structures for the AHL regulators require an updated review of the AHL-QS system. In this review, we provide a detailed overview of the AHL-QS system, the mechanism of AHL signal biomolecule synthesis, structural and functional analysis of the AHL synthase, and AHL receptors of Gram-negative bacteria, including *Pseudomonas aeruginosa*, *Chromobacterium violaceum*, *Agrobacterium tumefaciens*, and *Escherichia coli*. AHL signaling has been discovered in a wide range of bacteria, indicating that it is a widely accepted chemical language among Gram-negative bacteria for their adaptation and survival. Furthermore, biomolecules derived from bacteria can potentially be used for various applications, such as human and plant health, biofouling, bioremediation, biosensors, and cancer.

## 2. AHL Signal

**Figure 1:**
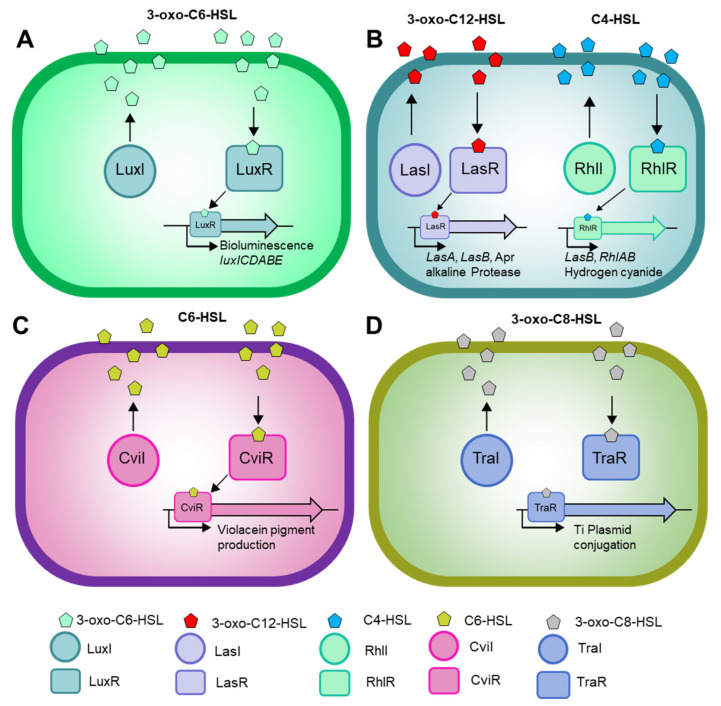
Overview of AHL-QS systems in Gram-negative bacteria. (**A**) Lux QS system regulating bioluminescence in Vibrio fischeri. (**B**) Las and Rhl QS system of *Pseudomonas aeruginosa* regulating the virulence and biofilm formation. (**C**) Cvi QS system in *Chromobacterium violaceum* modulating violacein production. (**D**) Tra QS system controlling the Ti plasmid conjugation in *Agrobacterium tumefaciens*.

Gram-negative bacteria use AHL molecules to regulate gene expression in a population density-dependent manner (Figure 1) [4]. When AHL concentrations exceed the critical detection threshold, the population is directed to coordinate the expression of target genes for diverse metabolic activities. In this section, we cover the AHL signal diversity in bacteria.

### 2.1. AHL Diversity

**Figure 2:**
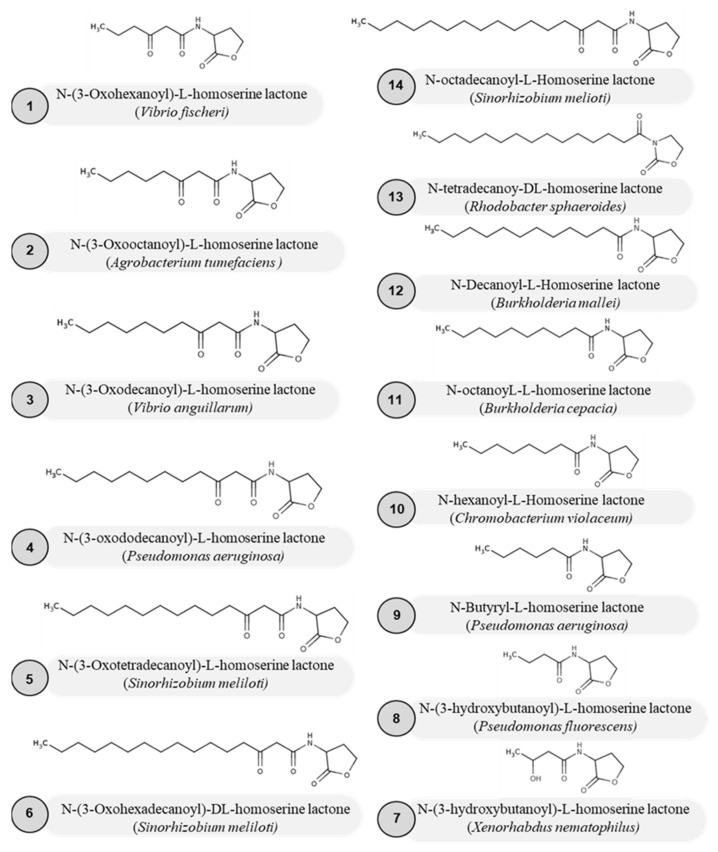
Structural diversity in AHL signals in bacteria. Chemical structure of various AHLs produced by bacteria: 3-oxo-C6-HSL1 (**1**), 3-oxo-C8-HS (**2**), 3-oxo-C10-HSL (**3**), 3-oxo-C12-HSL (**4**), 3-oxo-C14-HSL (**5**), 3-oxo-C16-HSL (**6**), 3-hydroxy-C4-HSL (**7**), 3-hydroxy-C8-HSL (**8**), C4-HSL (**9**), C6-HSL (**10**), C8-HSL (**11**), C10-HSL (**12**), C14-HSL (**13**), and C16-HSL (**14**).

Gram-negative bacteria produce a variety of AHL signals. The signal molecules have different acyl chain lengths, ranging from C4-HSL (*P. aeruginosa*) [19] to C18-HSL (*Sinorhizobium meliloti*) [20]. *P. aeruginosa* can synthesize both short-chain (C4) and long-chain (C12) AHLs from the same acyl-ACP reservoir [21]. *Aeromonas culicicola* produces AHLs with methylated acyl chains, and *Jannaschia helgolandensis* has AHLs with double bonds in the acyl chains [22]. AHLs are also distinguished based on their oxidation in the C3 position. Figure 2 shows the diversity in the AHL signals among various bacteria. We compiled a list of studies that report AHL diversity in diverse bacterial species that are discussed here. Yan et al. (2007) detected 3-oxo-C6-HSL in the culture supernatant of Vibrio fischeri using the bioreporter *E. coli* strain [23]. In *Yersinia enterocolitica,* LuxI produces the N-(3-oxo-hexanoyl)-L-homoserine lactone (3-oxo-C6-HSL) and the N-hexanoyl-L-homoserine lactone (C6-HSL) as major AHL molecules [24]. According to Morohoshi et al. (2019), 200 strains of *Pectobacterium carotovorum* subsp. *carotovorum* produced 3-oxo-C6-HSL as the primary AHL signal, whereas 70 strains produced 3-oxo-C6-HSL as the direct AHL signal [25]. TraI is a *Agrobacterium tumefacien* that produces the 3-oxo-C8-HSL autoinducer signal. The LuxRI QS system of *Vibrio anguillarum* has been found to produce 3-oxo-C10-HSL [24]. *P. aeruginosa,* LasI, produces a 3-oxo-dodecanoyl homoserine lactone that regulates virulence [26,27]. Long-chain AHLs such as 3-oxotetradecanoyl HSL and 3-oxohexadecenoyl HSL have been reported to be produced by the *sinRI* locus in *Sinorhizobium meliloti*. In the insect pathogen *Xenorhabdus nematophilus*, the N-(3-hydroxybutanoyl)-L-homoserine lactone molecule modulates virulence [28]. To control phenazine synthesis, *Pseudomonas fluorescens* produces N-(3-hydroxybutanoyl)-L-homoserine lactone molecules [29]. An additional QS has been well studied in *P. aeruginosa*, where RhlI produces C4-HSL as the signal molecule that is crucial for rhamnolipid synthesis [30]. Short-chain AHL molecules, such as C4-C8 HSLs, are produced by *Chromobacterium violaceum* strains [31,32]. CepI produces C8-HSL with low amounts of C6-HSL in *Burkholderia cepacia* [33,34]. C8-HSL and C10-HSL are synthesized in *Burkholderia mallei* to modulate virulence [34,35]. In *Rhodobacter* sphaeroides, CerR-CerI QS produced an unusually long-chain AHL signal (the N-tetradecanoy-DL-homoserine lactone) [36,37]. The AHL diversity reflects the structural and functional variation of AHL synthase proteins and their complex substrate recognition mechanisms. The mechanism of AHL synthesis and structural and functional studies of AHL synthases are discussed in the next section.

### 2.2. AHL Production

The AHL communication system is incredibly diverse among Gram-negative bacteria and is known to produce a wide range of AHL signals. The production and diversity of AHL molecules are governed by the AHL synthase enzyme [38,39]. The genome sequencing of diverse bacteria indicated the existence of numerous AHL synthesis protein biomolecules in a bacterial population [40,41,42]. Bioassay detection approaches have also proven the presence of AHL molecules in bacterial culture extracts [43,44]. The diversity among AHL signals is also linked to the type and expression level of AHL synthase enzymes. LuxI, HdtS, and LuxM biomolecules are critical families of the AHL synthase that have been studied in detail. To date, more than 90 species of bacteria are known to produce AHL molecules as their QS signal [45]. The type and the expression level of AHL synthase enzymes are linked with the diversity in the AHL signal production. At the molecular level, the control of AHL synthases is multifaceted, highly complex, and yet poorly understood.

### 2.3. AHL Synthesis Protein Biomolecules

LuxI-type protein biomolecules were discovered in *V. fisheri* and named after the origin or phenotype that they control [46,47]. LuxI-synthase homologs are found in numerous proteobacteria and are diverse in terms of their mode of AHL synthesis. LuxI-type synthase enzymes use S-adenosyl-L-methionine (SAM) and acyl-acyl carrier protein (acyl-ACP) to synthesize AHL [48]. The polar interaction between acyl-ACP and the amino acids of the substrate-binding site of the AHL synthase governs the substitution of the C3 position [38]. Research showed that threonine in the active site governs this interaction as a mutation in the residue to alanine, causing an increase in the production of 3-oxo-AHLs [49]. The amino acid diversity in the binding pockets plays a crucial role in forming various AHL signals. The correlation between the sequence and three-dimensional structure of the AHL synthase, as well as the acyl chain length of the substrate, were unable to provide the mechanism of diversity in AHL production.

The most conserved regions in the AHL synthase protein sequences are located in the first 100 amino acids of the N-terminal region [18]. SAM binding and catalysis are the key functions of this region. The C-terminal region is less conserved and is involved in the interaction and binding of acyl-ACP with its acyl chain. The sequence diversity in this region is justified by the heterogeneity in the acyl chain length of the AHL molecules. However, the precise mechanism of interaction is yet unclear. Most AHL synthases have the exact AHL synthesis mechanism; however, their substrate interaction mechanism and identification differ depending on the quantity of acyl-ACP substrates. The availability of LuxI crystallographic structures has paved the way for a better understanding of substrate–enzyme interactions at the molecular level [14,50]. However, the crystal structure of the AHL synthase coupled to the acyl-ACP complex has not yet been determined, which limits our ability to comprehend the interaction details.

**Figure 3:**
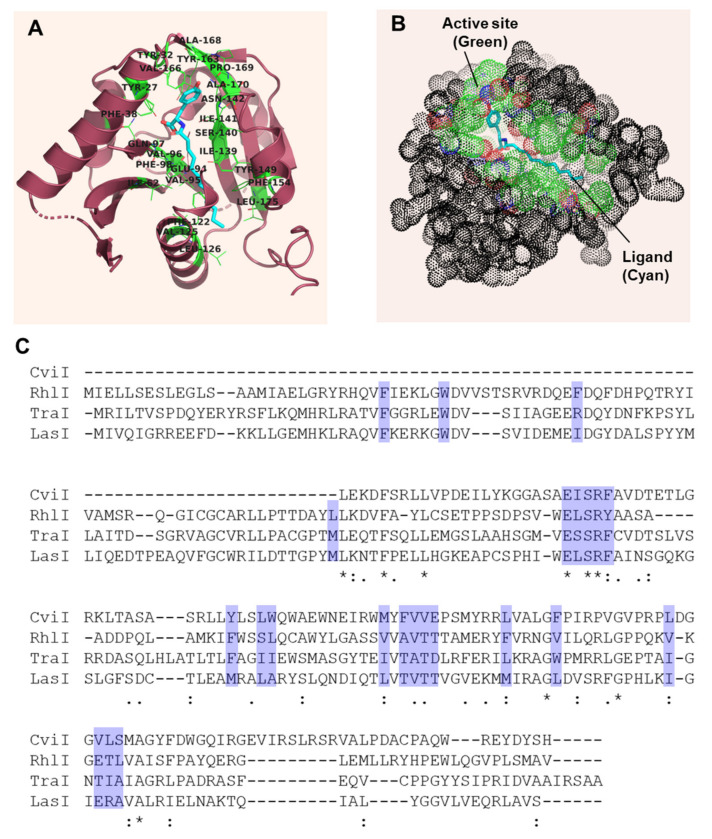
(**A**). Structural and sequence analysis of FeeM and AHL synthase proteins. Detailed analysis the ligand-binding pocket of N-acyl amino acid synthase (FeeM) PDB-ID:2G0B. (B). The dot representation of the protein with the ligand-binding site is highlighted in green with the ligand molecule (N-dodecanoyl-L-tyrosine) (**C**). Multiple-sequence alignments of the amino acid sequence of AHL synthase proteins (CviI, RhlI, TraI, and LasI) with highlighted regions (blue) correspond to the ligand-binding pockets of the FeeM.

Furthermore, GCN5-related histone nacetyltransferases (GNATs) [51] and acyl-ACP-dependent amino acid acyltransferase are remarkably similar to the AHL synthase (FeeM). This provides circumstantial evidence for interactions of AHL synthases with these substrates. Churchill et al. (2011) analyzed the sequences of LasI and EsaI and created a comprehensive interaction map between the enzymes and their substrates [18]. We analyzed the FeeM active site to determine its binding pocket. The availability of the substrate bound to the active site provided us with a detailed map of the residues in the active site (Table 1 and Figure 3A). The active site was buried deep (green color) in the enzyme structure, as shown in the Figure 3B (the dot representation of the FeeM shows the ligand and the active site). FeeM shows high structural similarity with AHL synthase homologs but lacks sequence similarity, limiting our ability to use multiple-sequence alignment tools to map the AHL homologs. Therefore, we first rendered the LasI on FeeM using the Swiss model and produced a model for our analysis. We used the LasI protein model to compare with the FeeM structure and mapped the homologous residues in LasI that are found to interact with the ligand. The 3D models of the AHL synthase proteins were constructed using the Swiss model, and their active sites were compared and analyzed.

#### 2.3.1. LasI

**Figure 4:**
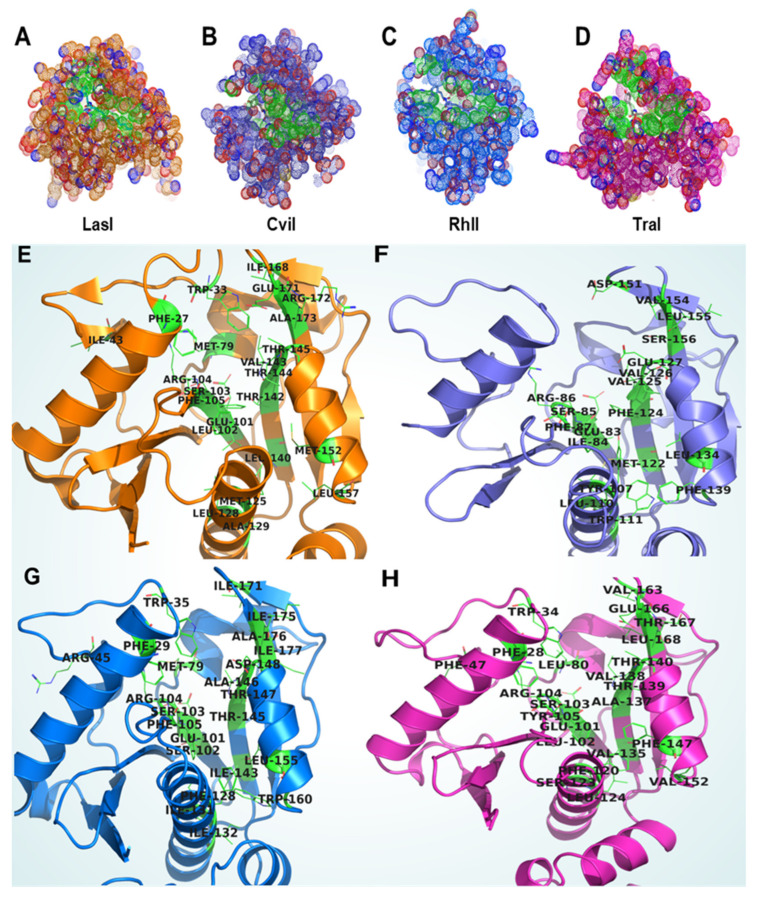
Detailed map of AHL synthases active sites. The dot representation of the protein highlights the active site (green) of AHL synthase proteins (LasI, CviI, RhlI, and TraI) (**A**–**D**). Detailed map of the residues of binding pocket in LasI (**E**), CviI (**F**), RhlI (**G**), and TraI (**H**).

LasI, an AHL synthesizing biological macromolecule of *P. aeruginosa*, was named after the induction of the elastase enzyme, which is a potent virulence factor in *P. aeruginosa* [17]. LasI produces 3-oxo-C12-HSL during the early development phases of *P. aeruginosa* growth. We mapped the residues in LasI that may be involved in the substrate-binding pocket in LasI using the 3D model. The LasI binding pocket was predicted and analyzed (Table 1 and Figure 4A,E). The active site in the LasI enzyme was also found to be buried deep and its position was very similar to the FeeM active site. To further validate our analysis, we performed multiple-sequence alignments of AHL synthase proteins and highlighted the residues of the active site (Figure 3C). Our study showed that many residues were conserved within the first 100 amino acids of the protein, and high sequence diversity was found towards the c terminal.

#### 2.3.2. RhlI

RhlI is involved in the synthesis of C4-HSL, and its expression has been observed in biofilms produced by clinically relevant *P. aeruginosa* strains [52]. As a result, this protein plays a critical regulatory role in *P. aeruginosa* pathogenicity. Gene expression studies showed that the upstream regulatory regions are a crucial regulator for RhlI expression, and rhl and las both regulate rhlI expression in *P. aeruginosa* [53]. Wild-type *P. aeruginosa*, with a functional QS system, was a more attractive prey for macrophages than the lasI/rhlI mutant [54]. Expression studies revealed that this regulatory region is vital for *rhlI* expression, and that las is a dominant regulator, even though the rhl QS system can induce *rhlI*. RhlI is controlled by the transcriptional regulator LasR, and its expression also drives LasI expression, resulting in the substantial amplification of 3-oxo-C12-HSL in the early phases of development. The Las and Rhl QS systems command their genes, expressed differentially at distinct stages of biofilm development [55]. In addition, AHL synthesis is directly influenced by the different concentrations, classes, and substrates of synthases across various stages of bacterial development [56]. It is clear from the structural analysis that the RhlI has the active site buried deep in the protein center and is comparable to the LasI site (Table 1 and Figure 4C,G). However, there is a significant variation among the amino acid residues of LasI and RhlI. This amino acid variation in the active site may contribute to the AHL diversity in *P. aeruginosa.*

#### 2.3.3. CviI

The *C. violaceum* QS system has CviI/CviR as the AHL synthase and AHL receptor biomolecules. Studies have shown that the QS CviI synthesizes the C6-homoserine lactone (HSL) that binds with CviR, leading to the regulation of vio operon (*vioA, vioB, vioC, VioD,* and *VioE*) [57]. The CviI binding pocket revealed a comparable binding pocket buried deep inside the protein (Table 1 and Figure 4B,F). The active-site residues differed from the majority of the AHL synthase proteins, demonstrating that diversity plays an essential role in AHL production CviI. There is a considerable gap in understanding CviI functions; hence, further research on the structural and functional features of the CviI/CviR QS system is needed.

#### 2.3.4. TraI

In *A. tumefaciens,* TraI governs the formation of the 3-oxo-C8-homo serine lactone, which binds to TraR and regulates TraI expression [15]. TraI/R QS, which is controlled by Ti plasmid transfer, governs the formation of crown galls in several plant hosts [58]. *A. tumefaciens* also have anti-activation mechanisms that prevent the QS response under low population densities [59]. The QS signaling suppression mechanism is crucial to take advantage of the cellular energy supply throughout their development cycle. In *A. tumefaciens*, AHL synthase (TraI) and anti-activator protein (TraM) expression is regulated by the TraR-AHL complex [47,60]. Novel anti-activator proteins might help us better understand QS regulation at the molecular level. Active-site analysis clearly shows that the site is buried deep in all the AHL synthase homologs and present in the middle occupying the center of the protein, as shown in the dot representation of the proteins (Table 1 and Figure 4D,H).

Our analysis may help establish the role of the active-site residues in determining the type of AHL formation. The data may also be used in silico molecular docking, as this would be a valuable drug binding site for pathogenic organisms such as *Pseudomonas aeruginosa.* We also observed that the enzymes are present in different confirmations; thereby, the role of molecular dynamics may also play an essential role in the synthesis of AHLs of variable lengths.

## 3. AHL-Dependent Transcription Regulator Biomolecules

AHL receptors or AHL transcriptional regulators bind directly with the corresponding AHL signal. As a result, the AHL receptor undergoes conformational changes, allowing it to connect to the target DNA molecule. The structure and function of the AHL receptor are thoroughly examined in this section.

### 3.1. LasR

*Pseudomonas aeruginosa* possesses many two-component transcriptional regulatory proteins that allow it to respond to environmental changes [61,62,63,64]. LasR is a crucial transcriptional regulator of *P. aeruginosa* that has been widely explored in terms of its virulence expression and biofilm formation [65,66,67,68,69,70,71,72,73,74,75]. The functional aspects of LasR have received significant attention; however, structural investigations have been sparse. The las QS system uses the N-(3-oxo-dodecanoyl)-L-homoserine lactone (OdDHL) as a chemical signal, which binds with the LasR protein [1]. As explained earlier, the LasI protein produces OdDHL, which binds to the LasR transcription regulator and induces downstream gene expression. When OdDHL binds to LasR, a conformational change occurs, and the activated LasR-OdDHL complex binds to las boxes (lux-box-like sequences) [76]. In the presence of OdDHL, LasR forms a multimer that can attach to the las boxes. By establishing a feedback loop, LasR also controls LasI expression. LasR/OdDHL also controls RhlR expression, resulting in signal amplification [77]. LasR and RhlR bind to lux-box-like sequences (las boxes), regulated by either LasR, RhlR, or both [78]. LuxR-type protein regulation is susceptible to relative protein concentrations; slight alterations can substantially influence gene expression. The *lasR* gene is expressed throughout the development cycle, with greater levels in the early stationary phase and lower levels in the late stationary phase. Although LasR mutations are frequent in chronic *P. aeruginosa* infections, it has been shown that these mutants preserve LasR function, with just a few being able to unlink the las and rhl systems [79]. Furthermore, environmental factors such as as iron and phosphate play a key role in the control of virulence through the las and rhl QS systems [80].

The Las system also works in conjunction with related regulatory proteins. RsaL, a key regulator of LasI, is also activated by LasR and controls OdDHL levels, pyocyanin, and cyanide synthesis through the bidirectional repression of *rsaL-lasI* genes [81,82]. In *P. aeruginosa*, the LasR protein has been linked to several regulatory mechanisms that control virulence and biofilm formation. LasR also regulates VqsR, a positive regulator of Las QS [30]. Another regulatory protein, QsIA, limits LasR activity by preventing it from interacting with the target DNA sequence through protein–protein interaction, independent of OdDHL/LasR concentrations. Vfr (CRP homologue) activates LasR, and GacA modulates the las and rhl systems [83]. Recent research has revealed a connection between LasR and the transcriptional factors Anr and Mhr [84]. Researchers have found that in low-oxygen settings, both LasR+ and LasR- strains require Anr and Mhr to produce biofilms. Recent studies have shown a relationship between the transcriptional factors LasR, Anr, and Mhr. LasR+ and LasR- strains need Anr and Mhr to produce biofilms in low-oxygen environments [84]. Furthermore, Anr activity induces the enhanced production of the oxygen-binding protein Mhr, offering lasR mutants an advantage in low-oxygen conditions. The relevance of LasR regulation has been shown by several in vivo investigations. A LasR-negative mutant was reported to be less pathogenic in a neonatal mouse model of acute pneumonia. In another study, *lasR*, *lasI*, and *rhlI* mutant strains diminished pathogenicity in a burn wound mouse model [85]. LasR mutants also demonstrated lower pathogenicity in the *Arabidopsis* nematode [86]. These findings imply that lasR-dependent virulence factor expression is critical for the establishment of *P. aeruginosa* infection.

**Figure 5:**
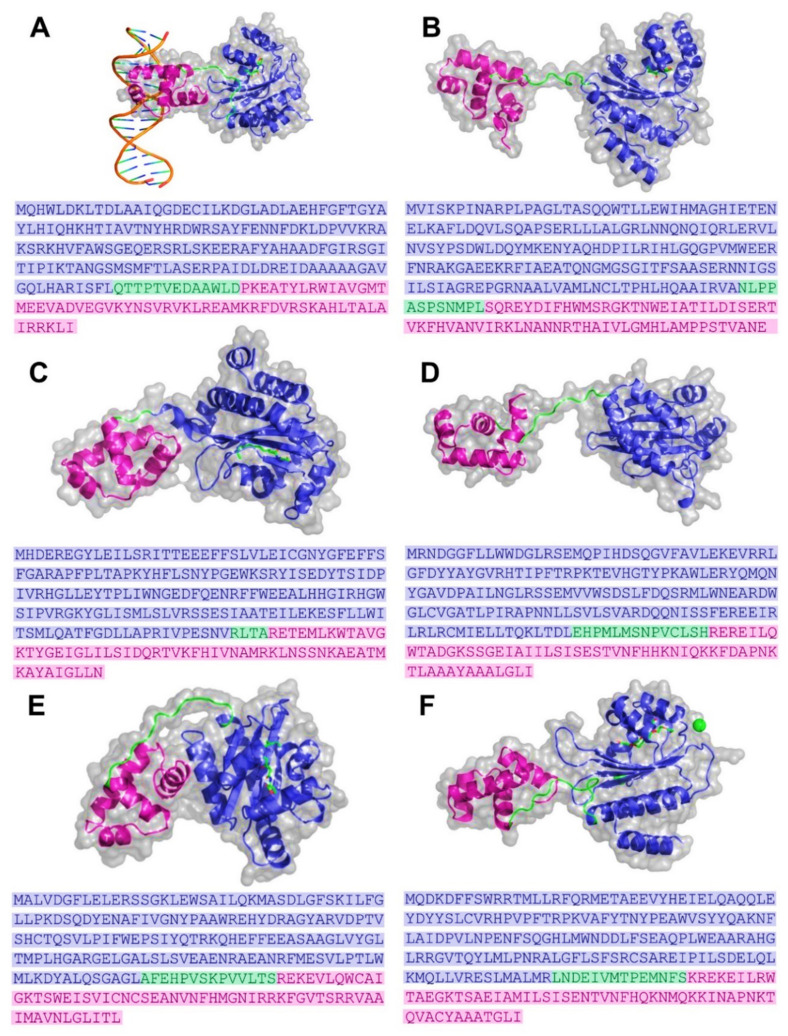
Structure of AHL regulators. Cartoon representation showing the ligand-binding domain (slate), domain-connecting loop (green), and the DNA-binding domain (DBD) of bacterial AHL regulators. The lower panel shows the FASTA sequence of the protein, highlighting the residues of the corresponding regions: (**A**) TraR, (**B**) CviR, (**C**) QscR, (**D**) RhlR, (**E**) LasR, and (**F**) SdiA.

**Figure 6:**
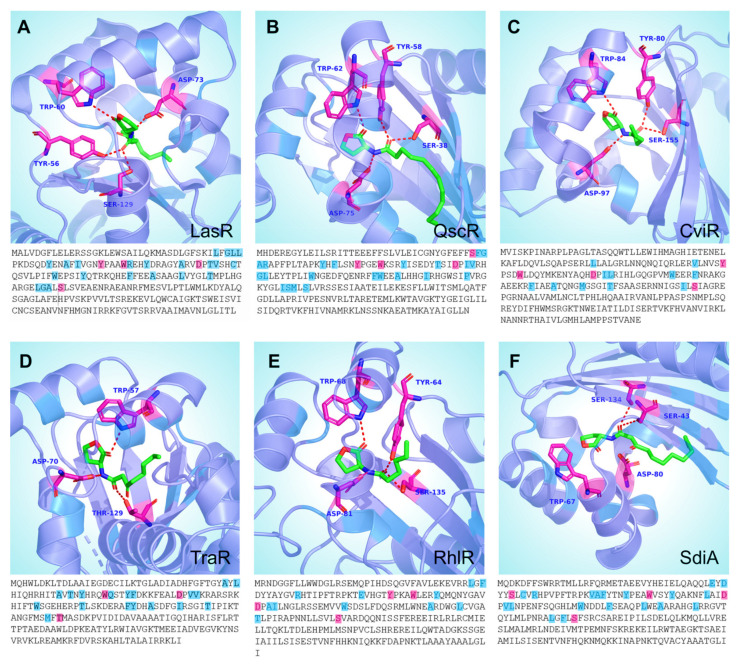
Ligand-binding site analysis of quorum-sensing regulators. The representation of cartoon sticks shows the contact residues of the ligand-binding site and the FASTA sequence highlighting the residues involved in ligand interactions (magenta: the h-bond-forming residues, blue: the residues within 5A of the ligand). The receptors are highlighted in slate: (**A**) LasR, (**B**) QscR, (**C**) CviR, (**D**) TraR, (**E**) RhlR, and (**F**) SdiA.

A structural investigation of the AHL transcription regulator revealed that it has three unique domains or regions: (a) the ligand-binding domain (LBD), (b) the connecting loop region, and (c) the DNA-binding domain (DBD). Although the LBD was more prominent than the DBD, the length of the loop region differed between the proteins. Structure/sequence information is provided in Figure 5A–F. A full-length crystal structure of LasR was available (PDB-ID: 6V7X). Structural analysis revealed a domain profile that was similar to that of other QS transcriptional regulators (Figure 5). In this structure, the autoinducer molecule (the N-3-oxo-dodecanoyl-lhomoserine lactone) and a phage-encoded anti-activator protein (Aqs1) of *Pseudomonas* phage DMS3 were both linked to the LasR protein. The Aqs1 protein was bound to the DBD. The ligand-binding site analysis of LasR showed that the ligand makes polar contact with Trp60, Asp73, Tyr56, and Ser129 (Figure 6A). These residues are highlighted in the FASTA sequence in blue (Figure 5A).

### 3.2. RhlR

The las and rhl QS systems function closely together, and findings reveal that the las and rhl systems control regulons with the overlapping gene sets. It is still unclear which parameters are responsible for the distinct preferences of promotors for various QS regulatory proteins. Studies have shown that the Rhl system regulates elastase, rhamnolipid, pyocyanin, and hydrogen cyanide production in *P. aeruginosa* (Table 1) [67,87,88,89,90]. Other studies have revealed that LasR, RhlR, and ANR (anaerobic regulator) synergized in the *hcn* promotor activation. Researchers also showed that even in the presence of RhlR and AHLs, the expression of *rhlAB* is not induced during the log phase of bacterial growth [91,92]. According to research utilizing *lasI* and *RhlI* mutants, both classes of AHLs (OdDHL and BHL) can substantially increase the expression of *P. aeruginosa* genes. However, it is difficult to access the role of each system by eliminating the other, since a defect is one system may affect the function of the other QS system. RhlR forms dimer and can bind to the las boxes in the presence and absence of C4-AHL [93]. To date, the crystal structure of the RhlR transcription regulator is still unsolved. The crystal structure is needed to provide accurate details of the RhlR domains. We used the Swiss model to build its model, using CviR (3qp5) as the template. The homology model was further analyzed for its structural features (Figure 5D). The homology model analysis showed three distinct domains (similar to other AHL regulators), a large LBD (Trp11 Leu169), a small loop (Glu170-His183), and a small DBD (Arg184-Ile241) (Figure 5D). The sequence identity of the model was 25.2% with a QMEAN score of −4.85. We analyzed the active site of RhlR (homology model) using BHL as a ligand. BHL showed polar contact interactions with Trp68, Tyr64, Ser135, and Asp81 (Figure 6E).

### 3.3. QscR

Additional LuxR-type members lacking cognate LuxI homologs were identified in the genome of *P. aeruginosa.* The newly found LuxR homolog was designated as QscR, and this system was revealed to be implicated in the deregulation of several AHL-QS-mediated virulence features [94]. At lower culture densities, the *qscR* mutant overproduced pyocyanin, elastase, and hydrogen cyanide compared to wild-type bacteria [95,96,97]. The QscR protein inhibits the production of 3-oxo-C12-AHL, and since 3-oxo-C12-AHL triggers the RhlI-RhlR system, it also suppresses the RhlI QS system. According to the mechanism of QscR suppression elucidated using fluorescence anisotropy, the formation of heterodimers between QscR and LasR, or RhlR, may result in the inhibition of AHL-LasR/RhlR interaction. The enhanced QscR-AHL interaction might be due to the elevated concentration of AHL. According to the current QscR function hypothesis, inactive heterodimers form at low AHL concentrations, blocking lasIR- and rhlIR-mediated QS signaling. However, there is an upsurge in AHL production during the early stationary phase, shifting the equilibrium to LasR-AHL and RhlR-AHL interactions, allowing for lasIR/rhlIR-dependent gene expression. The QscR crystal structure (6cc0) showed similar structural similarities with the other AHL transcriptional regulators, a large LBD (Arg1 to Val174), a short loop region (Arg175 to Thr177), and a small DBD (Ala178 to Asn237) (Figure 5C). The crystal structure analysis showed that the QscR is bound to its autoinducer at the ligand-binding site in the LBD [98]. The N-3-oxo-dodecanoyl-l-homoserine lactone is the ligand (autoinducer) used for QscR, and the ligand interaction revealed that it formed polar interactions with Trp62, Asp75, Tyr58, and Ser38 (Figure 6B).

### 3.4. TraR

In *A. tumefaciens*, the TraR QS receptor shows comparable ligand–protein interactions to QscR, LasR, and RhlR of *P. aeruginosa*. The TraI/R–3-oxo-C8-AHL-dependent QS mechanism regulates Ti plasmid conjugal transfer in *A. tumefaciens* [99]. TraM and TrlR proteins bind to TraR and form inactive complexes, limiting its activity. TrlR is similar to TraR (88% similarity with the first 181 amino acids) and appears to be a truncated version of TraR. The TraR-AHL complex forms dimer and binds to a specific nucleotide sequence (18 nucleotide), called *tra* boxes, to activate the receptors [99,100]. The ligand-binding domain of TraR is sufficient for AHL binding and dimerization. The DNA-binding domain of TraR has helix-turn-helix DNA-binding regions with a striking resemblance to various bacterial regulators. TraR can activate class-I- and class-II-type promoters, and D10 and G123 have been identified as crucial residues for TraR activation [101]. Furthermore, single-substitution mutant studies showed that W184, V187, K189, E193Q, V197, and D217 are defective in transcription [102]. The role of this region was suggested in RNA polymerase and TraR-mediated promotor activation. Wetzel et al. (2019) provided evidence that the additional regulatory genes *mrtR* and *tmsP* are involved in TraR regulation [103]. Six nucleotides in the *tra* box that make no direct contact with TraR may be required for high-affinity binding by facilitating a previous DNA bend, as per the structural analysis of TraR and DNA complexes [104]. Mutation analysis showed that Asp-10 and Gly-123 are crucial for Rpo (DNA-dependent RNA polymerase) interactions [105]. The autoinducer binding also increases DNA binding with the TraR protein [106]. Furthermore, it was shown that TraR increases the copy number of Ti plasmid via up to 250-fold increase in *repABC* operons [107]. Qin et al. (2000) showed that, in the absence of an AHL signal, the TraR protein is localized into the inner membrane and AHL-TraR complex formation leads to the transportation of this complex in the cytoplasm [108]. MD simulation studies showed the dynamic changes in the ligand–protein interaction in the ligand-binding domain and DNA-binding domains [109]. Relaxosome formation in Ti plasmid conjugation is governed by TraR-regulated proteins, including TraA, TraC, and TraD [110]. It also regulates mating pair formation during conjugation mediated by *trb* genes (type IV secretory system) [111]. The TraR protein also controls the expression of conjugation transfer proteins (TrbJ and TrbK), which regulates the exclusion mechanism during conjugation events [112]. These interactions may play a significant role in the DNA-binding ability of the protein. Many proteins often require chaperons for folding, e.g., TraR shows increased solubility and transcription in the presence of GroESL and its cognate AHL [113]. Anti-activator proteins in the cell are essential regulators of transcriptional factors, while TraM has been characterized to form an anti-activation complex that inhibits the binding of TraR with the target DNA [114]. In vitro and in vivo studies showed that the TraR-TraM anti-inactivation complex is the 151 kDa complex, formed by a two-step process. First, TraR interacts with the *tra* box-bound TraR protein and forms the 77 kDa complex, and further it forms 151 kDa species with concomitant DNA release [59]. Zhu et al. (1998) showed the potent inhibitor effect of a range of AHL analogs on the activity of the TraR protein [24]. These studies showed the importance of the protein structure and the binding mechanisms in understanding the activation process.

**Figure 7:**
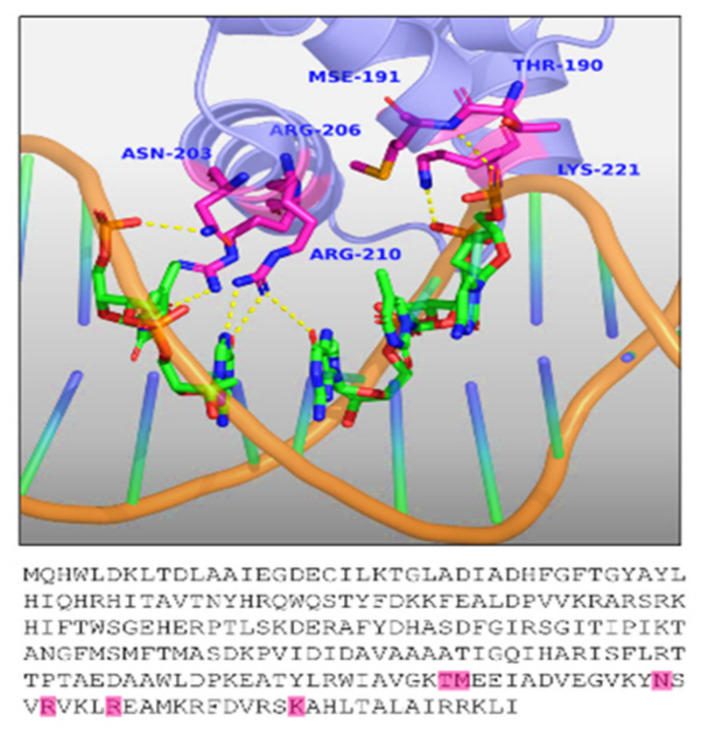
DNA-binding site analysis of TraR. The representation of cartoon sticks shows the contact residues of the DNA-binding site and the FASTA sequence highlights the residues involved in ligand interactions (magenta: h-bond-forming residues). The receptor is highlighted in slate with cartoon representation.

**Figure 8:**
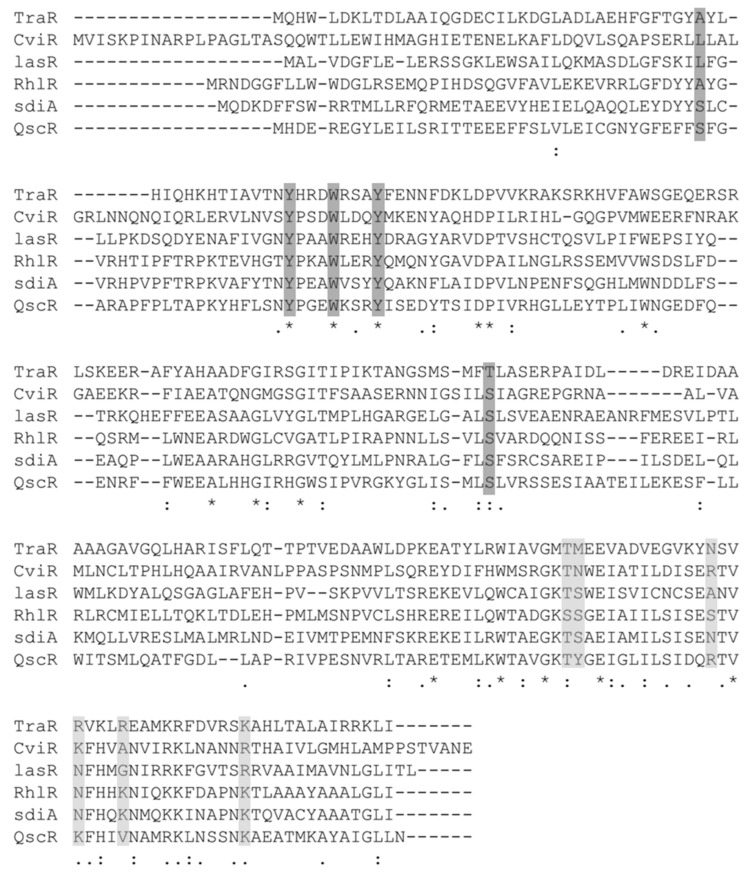
MSA analysis of QS regulators to compare the residue of the active site and the DNA-binding site. Sequence alignment of the QS regulator results in output using MUSCLE. Positions highlighted in dark gray are the residues involved in h-bond formation at the active site and those in light gray are the reference sites for DNA binding as per TraR structure information.

The TraR crystal structure (1H0M) is available with the bound autoinducer molecule (the N-(3-oxo-octanoyl)-L-homoserine lactone) and the cognate DNA molecule [115] (Figure 4A). The TraR LBD domain extends Met1 to Leu161, the loop region spans Arg162-Asp175, and the DBD spans Pro176 to Ile234 in length (Figure 5A). The N-3-oxo-dodecanoyl-l-homoserine lactone is the ligand for TraR and interacts with the residues Asp70, Trp57, and Thr129 of the ligand-binding domain (Figure 6D). The residues forming polar interactions with the target DNA were identified. Asn203, Arg210, Arg206, Lys221, Mse191, and Thr190 established polar interactions with the target DNA molecule (Figure 7). The polar contacts of the active site and the DNA-binding site in all transcriptional regulators were mapped using multiple-sequence alignment methods (Figure 8). Results showed that Thr190, Mse191, Asn203, Arg206, Arg220, and Lys221 are residues involved in polar contact formation with the DNA. Thr is present at this position for all transcriptional regulators, except for RhlR. In the RhlR protein, Thr is replaced by serine. At position 191, Mse is present in TraR; Asn in CviR; Tyr in QscR; and Ser in LasR, RhlR, and SdiA. Position 203 is occupied by Asn in TraR and SdiA, Arg in CviR and QscR, and Ala in LasR. At position 206, amino acids are present, Arg in TraR, Lys in CviR and QscR, LasR, RhlR and SdiA have Asn. Position 206 is occupied by Arg in TraR, Ala in CviR, Gly in LasR, K in RhlR and SdiA, and Val in QscR. Next, at position 221, Lys is present in TraR, RhlR, SdiA, and QscR, and Arg is present in LasR and CviR. Our analysis showed significant diversity among all AHL regulator proteins in the DBD of proteins. This is due to the specific cognate sequence in the genome. We carried out a similar analysis on the ligand-binding domains of these proteins. The crystal structure data showed that the Tyr53, Glu57, and Tyr61 in TraR play an essential role in ligand binding. Since most AHL molecules share a typical lactone ring structure, these three residues are conserved among all the proteins.

### 3.5. AHL Production

CviR is the cytoplasmic transcriptional regulator of *C. tumefaciens* and responds to C4, C6, C8, and C10-HSL molecules. Stauff et al. (2011) have shown that the CviR binds to a palindrome sequence CTGNCCNNNNGGNCAG [116]. The *C. violaceum* genome was scanned for putative CviR-binding sites. The analysis showed that CviR potentially regulates the expression of pigment, AHL synthase, chitinase, a type VI secretion-related gene, exoproteases, β-lactamase, histone deacetylase (antitumoral depsipeptide), and a guanine deaminase gene [116,117,118,119]. *C. violacein* (CV026), the mini-Tn5 mutant [120] (violacein-negative), has been extensively explored as a potential biosensor for the detection of AHLs and screening anti-QS inhibitors [121,122,123,124]. Ravichandran et al. (2018) have shown that the phytochemicals can interact with CviR, thereby inhibiting the expression of *cviI, cviR, vioB, vioC,* and *vioD* genes [125]. Structural analysis with ligand molecules and inhibitors showed that ligand binding at the native ligand-binding site could inhibit the protein, provided that the interaction could stabilize the closed confirmation [126]. The crystal structure (3qp6) of CviR revealed a conserved three-domain profile, including an LBD (Ile1 to Ala186), a short loop region (from Asn187 to Ser199), and a DBD (Gln200 to Asn264) (Figure 5B). The crystal structure was solved using the CviR protein of *Chromobacterium violaceum* 12,472 with its autoinducer molecule (the N-hexanoyl-L-homoserine lactone). C6-HSL is deeply embedded in the LBD and forms polar contacts with Asp97, Ser155, Trp84, and Tyr80 (Figure 6C).

### 3.6. SdiA

The genomes of *Escherichia* spp., *Salmonella* spp., and *Klebsiella* spp. lack luxI gene homologues, and these bacteria are often classified as non-AHL producers [127]. Most members of the Enterobacteriaceae family have a sole AHL-dependent transcriptional regulator protein, SdiA, which can sense and recognize AHL molecules synthesized by other microorganisms [128]. SdiA is a “suppressor of cell division inhibition” [129]. The SdiA protein controls gene expression via the SdiA-box, a critical regulatory protein located at the promotor regions of the gene. The SdiA-box contains the AAAA sequence (with minor variations) at the start and end regions with a spacer sequence in the middle, ranging from 8 to 18 nucleotides [130]. The SdiA protein modulates gene expression in the presence of natural AHLs (made by other bacteria) [127] and synthetic AHLs [131], and even in the absence of AHLs [132]. Furthermore, SdiA reacts to non-AHL ligands such as xylose and 1-octanoyl-rac-glycerol [132]. The role of SdiA has been linked with cell division, biofilm formation, motility, and antibiotic resistance [133,134,135,136]. In *E. coli,* the *ftsQAZ* operon in bacteria controls cell division via FtsQ, FtsA, and FtsZ proteins [128]. Two promotors control the regulation of the *ftsQAZ* operon, which is located upstream of the ftsQ gene and the reports suggested that the stationary-phase Sigma factor (RpoS) regulates promotor-1. Sdia regulates promotor-2, acting as a positive regulator for the ftsQAZ operon [137]. The SdiA protein of *E. coli* O157:H7 is activated by 1-octanoylrac-glycerol. The role of this protein has been proposed in various environmental conditions, including low-pH conditions. Ma et al. (1996) suggested the role of SdiA in acid tolerance by *E. coli* via GadW and GadY gene regulation [137]. GadW and GadY play an essential role in the acid resistance mechanisms of *E. coli.* Sperandio et al. (2010) have shown the role of SdiA in establishing the infection in the bovine rumen by sensing AHL signals [138]. By utilizing the CRISPR-Cas9 System, Askoura et al. (2000) have shown the role of SdiA in the cell adhesion, invasion, and biofilm formation by the Enteric pathogen, *Salmonella enterica.* Furthermore, their study also suggested that the SdiA mutation did not influence the intercellular survival of bacteria in HeLa and RAW264.7 cells [133]. A structural understanding of the SdiA protein is crucial in ligand interaction and gene expression activation. Grabski et al. (2021) functionally studied relevant confirmations of SdiA protein using molecular dynamic (MD) simulations and Markov modeling [139]. A structural comparison between the SdiA-acyl homo serine lactone and SdiA-1-octanoyl-rac-glycerol complexes provides crucial mechanistic insights into the ligand-dependent and -independent regulation of SdiA [132]. Yao et al. (2006) showed that the SdiA forms a complex with various AHL derivatives, proposing its role in signal detection from other bacterial species [130]. To provide a detailed mechanistic understanding of the SdiA protein, we analyzed the structure and the ligand-binding pocket, and also provided insights into the confirmational flexibility. The crystal structure of SdiA is available in PDB (4lfu), and structural analysis showed that it has a three-domain profile, similar to the previous AHL regulators. It has a large LDB (Asp5-Arg167), a loop (Leu168-Ser181), and a DBD (Lys182-Ile240) (Figure 4F). The C8-homoserine lactone molecule was used as the ligand for the purification of the SdiA protein. We analyzed the binding interaction of the ligand with the active-site analysis of SdiA. The results showed the polar contacts of the N-3-oxo-dodecanoyl-l-homoserine lactone with Trp67, Asp80, Ser43, and Ser134 (Figure 5F).

## 4. Applications of AHL-Dependent Quorum Sensing

Several clinically and industrially relevant microorganisms employ AHL-dependent QS to communicate and control phenotypic variations. The AHL-QS biomolecule is now being used to develop biosensor assays [140], anti-virulent compounds [141], and even anti-cancer therapeutics [142]. QS applications have thrived in agriculture, aquaculture, energy, bioremediation, and health research [143].

### 4.1. Human Health

The AHL-dependent QS systems allow gut microbes to interact with one another. AHL QS regulates bacterial phenotypic traits, which directly impact the host cell metabolism [144]. Lactobacillus and other proteobacteria are found in abundance in the human small intestine. These microorganisms employ AHL, AI-2, and other QS signals to control population density and prevent the development of gut infections. *P. aeruginosa* is a notorious bacterium responsible for a wide variety of illnesses [145]. *P. aeruginosa* regulates the expression of virulence factor synthesis and biofilm formation through AHL-dependent QS [146]. Novel techniques targeting *P. aeruginosa* QS may aid in developing novel anti-infective medicines against MDR *P. aeruginosa* infections. In addition, QS signal molecules have shown their potential role in cancer therapy. The *P. aeruginosa* QS signal (3-OC12HSL) has been shown to suppress the growth of human breast cell lines by causing apoptosis in cancer cells [147]. AHL homologs of natural and synthetic QS molecules may protect against infection and cancer. AHL molecules have shown potent immunomodulatory activity against immune cells [148], and the conjugation of AHLs with carrier proteins has been explored as a novel conjugate vaccine development strategy against *P. aeruginosa* infection [149]. AHL molecule detection in urine samples has been used as a diagnostic strategy against *P. aeruginosa*-dependent urinary tract infection [150]. Beneficial microorganisms or genetically modified microbes with AHL QS may be shown to have clinically significant effects on disease and cancer therapy. Plant extracts (garlic [151], ginger [152], and cranberry [153]) have shown QS inhibitory potential. Phytochemical-based QS inhibition has shown promising results as novel anti-QS and anti-biofilm agents. Zingerone [146], the villain [154], and curcumin [155] have shown anti-virulence effects against *P. aeruginosa* via targeting AHL QS pathways.

### 4.2. Controlling Plant Diseases

The overproduction of QS signals by epiphytic bacteria may result in QS suppression in *P. syringae* [156]. This might potentially inhibit swarming motility and infection prevention in tobacco plants [157]. Therefore, genetically engineered epiphytic bacteria may be employed as plant disease protective agents [158]. AHL-degrading microorganisms have a crucial function in combating plant diseases. The application of M. testaceum (AHL-degrading bacterium) protects against the plant pathogen *P. carotovorum* [159]. Quorum-quenching enzymes, such as acylases [160] and lactonases [161], have shown protective efficacy as anti-QS agents. A significant limitation of using these enzymes in animals is the allergic reactions and immune response against the proteins; however, in plants, these can provide high efficacy and applicability. The genetic engineering of plant or microbe genomes with QQ enzymes can be applied to control the concentration of AHL molecules and the pathogenic bacteria’s virulence. Lactonase (Aiia enzyme)-producing transgenic plants may resist *E. carotovora* infection in tobacco, potato, and cauliflower plants [162]. *M. tranculata* and *P. sativum* can produce structurally similar AHL compounds that mimic the AHL signal and interfere with their QS signaling pathway [163].

### 4.3. Plant Growth Promotion and Defense

Rhizobacteria employ AHL signals to regulate their growth and virulence. Plants can recognize AHLs at early stages of their expression [164]. Leguminous plant *Medicago truncatula* has been found to identify bacterial signal molecules. *M. truncatula* responded to 3-oxo-C14-HSL (*Ensifer meliloti*) and 3-oxo-C16-HSL (*P. aeruginosa*) [165]. The plant responded by inducing the genes associated with auxin-responsive and flavonoid biosynthesis pathways. Plants also expressed small signal molecules such as AHLs in response to bacterial AHLs disrupting the bacterial signaling system. The tomato plant, in response to C6- and C8-HSL-producing *Serratia liquefaciens* MG1 and *P. putida*, showed increased resistance against the fungal pathogen *Alternaria alternata* [166]. Bacteria induced the production of salicylic acid and ethylene-dependent defense genes (PR1a and chitinase). The protective effect was not detected with bacteria lacking the AHL signaling system. Verticillium dahlia-mediated wilt in *Brassica napus* was suppressed by rhizosphere bacterium *S. plymuthica* [167]. The effect was attributed to extracellular chitinase production mediated by AHL (C4-, C6-, and 3-oxo-C6-HSLs) and fungal inhibitory volatiles. Short-chain HSL molecules showed increased root length with variable phytohormones expressions in *A. thaliana* roots. Furthermore, the impact of long-chain AHLs (C14- and C12-HSL) significantly affects the biological control of the plant pathogen *P. syringe* in *A. thaliana* [168].

### 4.4. Biofouling

Biofouling the membrane filters by microbes limits the durability of the filtration system. This influences the membrane filtration performance and the destruction of the filters leading to the decreased performance and quality. Most biofilms are formed by *P. putida* and *Aeromonas hydrophila* [169]. Using QS inhibitors as membrane coating or using QQ enzymes expressing genetically engineered microbes may reduce the formation of biofilms on the membrane filters [170]. Yeon et al. (2010) showed that using an acylase-based membrane may minimize biofilm formation up to 24% [171]. *Rhodococcus* and the recombinant *E. coli* strain, capable of degrading AHL molecules, showed antibiofilm activity in wastewater treatment protocols [172]. Vanillin as an AHL signal activity modulator prevented biofilm in reverse osmosis (RO) apparatus on a cellulose acetate membrane filter [170]. Membrane biofouling prevention is an emerging field with promising applications in water purification and wastewater treatment strategies.

### 4.5. Bioremediation

Microbes play a central role in bioremediation by degrading toxic and harmful chemical compounds. QS has been shown to play an essential role in the degradation of many poisonous compounds, such as phenols [173], phenanthrene [174], hexadecane [175], and total organic carbon. Phenol is used in bulk quantities by industries (such as petroleum, tanneries, textile, dye, plastic, and agriculture) and hospitals (such as a disinfectant) [176]. Environmental phenol buildup harms human and animal health. Phenol may induce nervous system damage, renal dysfunction, corneal whitening, skin rash, dysphasia, gastric compilations, and cancer [177]. *Pseudomonas (P. putida, P. aeruginosa,* and *P. pictorum), Bacillus* (*Bacillus brevis*), *Arthrobacter (A. citreus* and *A. chlorophenolicus*), and *Cyanobacterium synechococcus* are known phenol-degrading microbes [178]. Phenol stress upregulates genes involved in cofactor biosynthesis, cell division, DNA repair and replication, heat shock proteins, fatty acid, and energy metabolism. *P. putida* phenol stressed fatty acid biosynthesis genes (FabB and FabH2) [179]. *P. putida* LPS biosynthesis genes (LpxC, MurA, and VacJ) and heat shock proteins (HtpG and GrpE) are also upregulated under phenol stress [180]. The AHL supplementation during wastewater treatment has enhanced phenol’s biodegradation for longer time intervals [181]. Δ*rhlI* and Δ*rhlR* mutant strains of *P. aeruginosa* have shown reduced phenol degradation capabilities compared to the wild-type strains [182]. *Acinetobacter* sp. showed increased hexadecane degradation by 3-OH-C12-HSL supplementation [183]. QS pathways also regulate the expression of the enzymes involved in bioremediation or biotransformation. The expression of catechol-1,2-dioxygenase, which is responsible for the biodegradation of anthranilate and phenol by *P. aeruginosa,* is enhanced by the exogenous supplementation of C4-HSL, C8-HSL, and C10-HSL [184]. The PQS-based QS system in *P. aeruginosa* regulates NO reductase, NO_3_^−^ reductase, and NO_2_^−^ reductase [185].

### 4.6. Biosensor Development

AHL-based biosensor development has been used for Gram-negative pathogen detection. AHL biosensors consist of AHL-responsive promoters, AHL receptors, and a reporter gene. The bio-detection of AHL has been linked to the indirect detection of pathogenic bacteria in clinical products, environmental samples, dairy products, meat products, and drinking water. AHL-based biosensors have been developed to detect *E. coli* O157:H7 [186]. AHL-based biosensors can be designed to detect Shiga toxin inputs from the bacteria. This input information can be processed via the AHL-based engineered QS circuit, and a reporter gene output can be produced. Lactic acid bacteria can be engineered to express reporter gene expression in the presence of gut microbes by utilizing the potential of QS circuits [187].

### 4.7. Cancer

3-oxo-C12-HSL, produced by *P. aeruginosa*, induces apoptosis in human breast cancer cells. However, its direct utilization is prohibited due to its immunomodulation, cell cytotoxicity, and hypervirulence activity in immunocompromised patients [188]. However, this may provide a chemical scaffold to develop novel anti-cancer drugs with targeted anti-cancer activities. Furthermore, tumors in vivo microbial communities may be exploited to design biosensors or therapeutics against cancer cells. The previous report has shown that the microbial population density can be used to invade cancer cells by engineered bacterial cells. This research uses the invasion gene (*Yersinia pestis*) by *E. coli* to invade cancer cells expressing the beta1-integrin receptor [189]. The threshold population density triggers the AHL production and causes an expression in the invasion and penetration of human cancer cells. Furthermore, *Bacillus*- and *Enterococcus faecium*-derived QS peptides invade and promote the angiogenesis of colon cancer cells, thereby promoting cancer cell metastasis [190,191]. At the molecular level, there is a need to explore the link between QS-derived bacterial phenotypes and colon cancer. In short, QS-based approaches can be used to develop biosensors and targeted drug delivery vehicles. The biggest challenge is controlling the immune response against such biosensors and the ability to distinguish between normal and cancer cells. Because of this, the primary exploratory site for developing such technologies is a colon, where microorganisms coexist closely with human cells. This allows bacteria adaption strategies to be developed for potential application, such as whole-cell biosensors, drug carriers, and treatment options for colon cancer and gut-associated diseases.

Overall, the QS regulatory systems details for the transcriptional regulators, associated virulence factors, and desirable functions is presented in Table 2.

## 5. Conclusion and the Future Prospects

AHL QS is necessary for bacteria adaptation, cellular growth, cell adhesion, biofilm development, cell division, antibiotic resistance, plasmid conjugation, and virulence gene expression in Gram-negative bacteria. Biological molecules such as AHL signals, AHL synthase, and AHL receptors have diverse structural and functional diversity across bacteria. The heterogeneity of the AHL-QS system may facilitate the specificity of intracellular and intercellular bacterial signaling systems. An understanding of molecular mechanisms and structural insights, particularly conformational dynamics, is crucial for understanding AHL-QS systems. Quantifying the conformational flexibility of proteins with and without ligands and inhibitors may reveal new insights into regulatory mechanisms. Currently, the role of confirmational dynamics and protein function is poorly understood. AHL transcriptional regulators can be employed as a model system to explore conformational change and its implications on gene expression. Furthermore, the findings of our structural analysis might be relevant in developing site-specific or allosteric inhibitors. Further research is required to comprehend how the AHL-QS system interacts with plant or animal host cells. The AHL-QS system could help researchers better understand the fundamental regulatory framework for gene expression in prokaryotes and aid in developing novel anti-virulence approaches as a future antimicrobial strategy and adaptation.

## Figures and Tables

**Table 1:** Proteins involved in the AHL substrate recognition, biological function, and their active-site residues.

Protein	Function	Active-Site Residue
FeeM	Acyl-ACP-dependent amino acid acyltransferase	Ala168, Try32, Tyr 163, Val166, Pro169, Tyr27, Ala170, Asn142, Phe38, Ile141, Ser140, Gln97, Val96, Ile139, Glu94, Tyr149, Val95, Phe154, Leu175, Phe122, Val125, and Leu126
LasI	3-oxo-C12-HSL production	Ile168, Trp33, Glu171, Arg172, Ala173, Phe27, Ile43, Met79, Thr145, Val143, Thr144, Arg104, Ser103, Thr142, Phe105, Glu101, Leu102, met152, Leu140, Met125, Leu128, and Ala129
RhlI	C4-HSL production	Ile171, Trp35, Ile175, Ala176, Ile177, Phe29, Ile177, Asp148, Met79, Arg45, Thr147, Thr145, Arg104, Ser103, Phe105, Glu101, Ser102, Leu155, Ile143, Phe128, Trp160, Ile131, and Ile132
CviI	C6-HSL production	Val154, Leu155, Ser156, Glu127, Val126, Val125, Arg86, Ser85, Phe124, Phe87, Glu83, Ile84, Met122, Leu134, Tyr107, Leu110, Phe139, and Trp111
TraI	3-oxo-C8-HSL detection	Val163, Glu166, Trp34, Thr167, Leu168, Thr140, Val138, Thr139, Ala137, Phe28, Leu80, Phe47, Arg104, Ser103, Tyr105, Glu101, Leu102, Val135, Val152, Phe120, Ser123, and Leu124
LasR	3-oxo-C12-HSL detection	Leu36, Gly38, Leu38, Leu38, Tyr47, Ala50, Ile52, Arg61, Tyr64, Ala70, Thr75, Val76, Cys79, Trp88, Tyr93, Phe101, Ala105, Leu110, Thr115, Leu125, Gly126, and Ala127.
RhlR	C4-HSL detection	Arg48, Glu59, Tyr72, Ala83, Ile84, Trp96, Ala111, Leu116, and Thr121
QscR	3-oxo-C12-HSL detection	Phe39, Gly40, Ala41, Arg42, Tyr52, Phe54, Tyr66, Thr72, Ile77, Val78, and Gly81 Leu82, Trp90, Phe101, Trp102, Ala105, Ile110, Pro117, Ile125, Ser126, Met127, and Ser129
TraR	3-oxo-C12-HSL detection	Ala38, Leu40, Ala49, Thr51, Gln58, Tyr61, Phe62, Val73, Val74, Trp85, Phe101, Tyr102, Ala105, Ile110, Thr115, Mse127, and Ala168
CviR	C6-HSL detection	Leu57, Val75, Leu85, Tyr88, Ile99, Leu100, Trp111, Phe115, Phe126, Ala130, Met135, Thr140, and Ile153
SdiA	3-oxo-C12-HSL detection	Cys45, Arg47, Val57, Ala58, Phe59, Tyr63, Tyr71, Leu77, Val72, Leu77, Trp95, Phe100, Leu106, Ala110, Leu115, Leu130, and Phe132

**Table 2:** The transcriptional regulators, associated virulence factors, and functions of QS regulatory systems.

QS Regulator	Phenotype/ Virulence Factor	Genetic Marker	Function	References
LasI/R	Protease	*lasA*	Epithelial barrier disruption, the adaptation and spread of infection, and immune evasion	[192]
	Elastase	*lasB*	The degradation of elastin, collagen, and related matrix proteins; the spread of infection; and extracellular iron acquisition	[193]
	Alkaline protease	*aprA*	The degradation of host proteins (complement, and cytokines), the establishment of infection, and immune evasion	[194]
	AHL synthase	*lasI*	Autoinducer expression	[195]
	Transcriptional activator protein	*Anr* and *Mhr*	Biofilm formation under low-oxygen conditions	[84]
RhlI/R	Rhamnolipids	*rhlAB*	The necrosis of host immune cells, biofilm formation, and immune evasion	[88,91]
	Pyocyanin	*phzABCDEFG* and *phzM*	Oxidative stress, inflammatory response, neutrophil toxicity, the establishment of infection, and damage to host cells	[196]
	Hcn hydrogen cyanide (RhlR)	*hcnABC*	Cell toxicity and infection establishment, lung cell damage, and poor lung function	[79]
	Autoinducer production	*lasI and rhlI*	The production of AHL molecules	[197]
QscR	Pyocyanin and hydrogen cyanide production	*phz* and *hcn*	Virulence factors, cell toxicity, and host cell damage	[95,96]
	Las/Rhl quorum-sensing-dependent genes	*lasIR* and *rhlIR* and associated genes	Quorum-sensing-dependent genes	[198]
TraI/R	Regulatory gene	*mrtR* and *tmsP*	Transcription regulation	[103]
	Conjugative transfer protein	*TraA, TraC,* and *TraD*	Conjugation	[110]
	Type IV secretion family	*trb* genes	The transfer of Ti plasmid	[111]
	Conjugative transfer protein	*trbJ* and *trbK*	Conjugation	[112]
CviI/R	Pigment production	*vioA, vioB, vioC, vioD*	Violacein pigment production, known for its antioxidant properties	[116,125]
	AHL synthesis and detection	*cviI cviI* and *cviR*	AHL synthase activity to enhance the production of AHL molecules via a positive feedback loop	[116,125]
	Chitinase production	Chitinase genes	Degrade chitin	[119]
SdiA	Cell division	*ftsQ, ftsA* and *ftsZ*	The positive regulation of the ftsQAZ gene cluster	[129]
	Acid tolerance	*GadW*, and *GadY*	Acid tolerance in *E. coli*	[137]
	Cell attachment and biofilm formation	*rck* and *srgE* locus	Enhanced cell adhesion, invasion, and biofilm formation by enteric pathogen *Salmonella enterica*	[133,199]
	Bacterial motility	fliC and csgA	The repression of flagella and curli fimbriae	[134]

## Data Availability

Not applicable.
